# How do slums change the relationship between urbanization and the carbon intensity of well-being?

**DOI:** 10.1371/journal.pone.0189024

**Published:** 2017-12-08

**Authors:** Julius Alexander McGee, Christina Ergas, Patrick Trent Greiner, Matthew Thomas Clement

**Affiliations:** 1 Department of Sociology, Portland State University, Portland, Oregon, United States of America; 2 Department of Sociology, University of Tennessee, Knoxville, Tennessee, United States of America; 3 Department of Sociology, University of Oregon, Eugene, Oregon, United States of America; 4 Department of Sociology, Texas State University, San Marcos, Texas, United States of America; Mercator Research Institute on Global Commons and Climate Change, GERMANY

## Abstract

This study examines how the relationship between urbanization (measured as the percentage of total population living in urban areas) and the carbon intensity of well-being (CIWB) (measured as a ratio of carbon dioxide emissions and life expectancy) in most nations from 1960–2013 varies based on the economic context and whereabouts of a substantial portion of a nation’s urban population. To accomplish this, we use the United Nations’ (UN) definition of slum households to identify developing countries that have substantial slum populations, and estimate a Prais-Winsten regression model with panel-corrected standard errors (PCSE), allowing for disturbances that are heteroskedastic and contemporaneously correlated across panels. Our findings indicate that the rate of increase in CIWB for countries without substantial slum populations begins to slow down at higher levels of urbanization, however, the association between urbanization and CIWB is much smaller in countries with substantial slum populations. Overall, while urbanization is associated with increases in CIWB, the relationship between urban development and CIWB is vastly different in developed nations without slums than in under-developed nations with slums.

## Introduction

Previous research has demonstrated that increases in urban slum populations in developing nations are associated with increases in infant and child mortality rates, and with decreases in energy consumption [1]. An essential question in discussions pertaining to climate change and appropriate mitigation policies is the extent to which carbon dioxide emissions can be reduced without sacrificing the well-being of individuals [2, 3, 4]. A recent body of research has emerged that assesses the relationship between various forms of socioeconomic development and what is known as the carbon intensity of well-being (CIWB), measured as a ratio of carbon dioxide emissions/life expectancy at birth. The goal of these studies is to analyze the extent to which socioeconomic development increases the well-being of individuals in nations and reduces anthropogenic contributions to climate change. Life expectancy at birth, is generally understood to be a good measure of human well-being (although it is by no means perfect), since it directly captures the health conditions of a society, such as infant mortality and life longevity, and indirectly reflects health processes such as prenatal education and high levels of literacy[5, 6]. Urban development is a crucial part of these discussions, as it has been found to increase and decrease both carbon dioxide emissions and well-being independently [7, 8, 9, 10, 11, 12, 13]. However, very little attention has been given to the relationship between urbanization (another key contributor to both rises in life expectancy and carbon dioxide emissions) and CIWB. Of the existing research exploring this relationship, it has been noted that a potential complicating factor in assessments pertaining to the correlation between urbanization and CIWB is the variation in forms of urban development cross nationally [6]. In this analysis, we address some of these concerns, as well as others pertaining to urban development and carbon dioxide emissions, by constructing a statistical model that assesses the nonlinear relationship between CIWB and urban population growth in developing countries that have considerable slum populations relative to urban growth in developed countries and/or countries with no slum populations. The goal of our research is to assess the broad implications of the relationship between urbanization and CIWB. Below we review relevant research on the topics of urbanization and CO_2_ emissions, urbanization and well-being, and CIWB to discuss how these works inform our theoretical and methodological approach.

## Previous research on urbanization, CO_2_ emissions, and well-being

Research on CIWB has developed out of macro-level quantitative research assessing the relationship between various forms of socioeconomic development and CO_2_ emissions. In these studies, urbanization is often observed as form of socioeconomic development. Liddle argues that macro-level quantitative research assessing the relationship between urbanization and CO_2_ emissions “comes in two different flavors” and can be distinguished between studies that assume the relationship between urbanization and CO_2_ is one-way causal, and studies that assume the relationship is multi-causal [14]. The latter of these two approaches employs the Granger-causality and vector error correction modeling technique and analyzes the short run effect of urbanization on CO_2_ emissions. However, these studies are mostly limited to exploring the relationship between urbanization and CO_2_ emissions in specific nations (the exception here being Mishra et al., Hossain [15] and Al-mulali et al. [16], thus this approach is not useful for our analysis as we explore the relationship between urbanization and CIWB in multiple nations over time. Since our methodological and theoretical approach falls more in line with the assumptions of one-way causal models, we will limit our review of relevant literature to such analyses.

In this tradition, studies have mostly found that the relationship between urbanization and CO_2_ emission is positive [17, 18, 19, 20, 21, 22] with a few notable exceptions. Liddle and Lung [23] found that urbanization, measured as the percent of individuals living in urban areas (the same measurement employed in our model) had an insignificant influence on total carbon emissions in OECD countries. In a similar vein, Jorgenson [24], Jorgenson [25], and Jorgenson and Clark [26] found that urbanization had a small influence on CO_2_ emissions. Liddle [27] also found that urbanization had a negative relationship with per capita road energy use in OECD countries.

Other studies analyzing the relationship between urbanization and CO_2_ emissions have explored the existence of an Environmental Kuznets curve, where the relationship between urbanization and CO_2_ emissions follows a nonlinear inverted-U shaped trajectory. York [16] tested for such a relationship but found that the quadratic term for urbanization was not significantly correlated to CO_2_ emissions. However, both Chun-yan et al. [28] and Martínez-Zarzoso et al. [29] found that urbanization has an attenuating relationship with CO_2_ emissions in both China and developing nations respectively, confirming what is known as the Environmental Kuznets Curve hypothesis [30].

To date, only one study [31] has explored the relationship between urbanization and life expectancy at birth. In this study, it was found that urbanization increases life expectancy at birth, although the relationship is quite small. Thus, based on existing research on the relationship between urbanization and CO_2_ emissions and life expectancy at birth, it has been found that urbanization increases both independently. Our study is concerned with urbanization as a form of sustainable development. Specifically, we are interested in whether urbanization increases life expectancy at birth at a rate that decouples the ratio of CO_2_ emissions and life expectancy at birth over time. With this in mind, we discuss previous research on CIWB and our conceptualization of the nature of urban development that informs our modeling approach in more detail below.

Most of the existing research on CIWB has been produced by Jorgenson and colleagues [12, 32, 33, 6]. In this research it has been found that a variety of socioeconomic indicators increase CIWB, specifically, economic growth, urbanization, and income inequality. Based on these analyses, it can be concluded that modern trends in socioeconomic development, such increases in GDP per capita, rises in urban development, and increases income inequality, result in rises in CIWB over time. All of these analyses use cross sectional data and employ the Prais-Winsten regression model with panel-corrected standard errors technique to assess the relationship between socioeconomic development and CIWB through time. The bulk of these studies use statistical interactions between time dummy codes and variables of interest to assess how the relationship changes over time [12, 34, 35, 6]. Additionally, research on CIWB has grouped nations based on their geographic proximity to one another or their economic and trade relations.

Our analysis is concerned with the nonlinear relationship between urbanization and CIWB in nations over time, specifically, at higher levels of urban development. As a result, we employ a quadratic term to our urbanization variable instead of using the time dummy interaction technique, which does not directly assess the strength or significance of the nonlinear relationship between urbanization and CIWB as urbanization increases. Additionally, due to our interest in the urban development that occurs in countries with substantial slum populations, we include an interaction term to assess the significance of this difference across all nations. As a result, we do not look at specific groups of nations separately but, instead, the significance of a specific group of nations in relation to all other nations where data is available.

## Conceptualizing urbanization

This research frames urbanization as a process that brings more people from the country to the city, which has multiple consequences in terms of carbon emissions (for review see Liddle [13]). These consequences can be summarized as follows. First, urbanization is associated with industrialization. The result of this process is that proportionately fewer people work in agriculture as the population shifts to urban areas, thereby accelerating fossil fuel use for construction and manufacturing purposes. Second, living in a city instead of a rural area entails not only a different mode of transportation but also higher levels of transportation activity, which, when combined, makes humans more reliant on the combustion of fossil fuel to move around in space. This is especially true in the developing world. Third, urbanization is associated with a decline in overall biomass fuel used for residential purposes and, as a result, as the population becomes more urban they tend to supplement or replace biomass with petroleum products [36].

## Logic of our model

The logic of our modeling approach is to control for known drivers of life expectancy at birth and CO_2_ emissions, and assess the specific relationship between increases in the percentage of urban populations and changes in CIWB. As mentioned previously, some studies have found that urbanization can have an attenuating relationship with carbon dioxide emissions [27, 28]. Specifically, this research has noted that the relationship between urban population and carbon dioxide emissions follows a nonlinear inverted-U shaped trajectory. To account for this potential nonlinear relationship between urban population and CIWB we include quadratic terms not only for our percent urban population variable but also for that variables′ interaction with a dummy measure that represents whether or not the country has a substantial slum population, a measure that we describe in greater detail below. As mentioned previously, a potential problem in assessing the relationship between growth in urban populations and CIWB is that urban development varies drastically across nations. While our modeling approach addresses these variations (see Methods section for more details), we still believe it is useful to understand the differences in the trajectories between nations that have clear distinctions in urban population growth. Thus we uniquely identified developing countries whose urban populations contain a substantial number of households facing slum conditions using the UN HABITAT and Millennial Development Goals indicators [5] (see Appendix 1). The UN identifies slum populations as “a group of individuals living under the same roof lacking ***one or more*** of the following conditions: access to improved water; access to improved sanitation; sufficient-living area; durability of housing; and security of tenure”. However, in practice the first four of these indicators are typically used in defining a slum household because secure tenure data are generally unavailable. We create an interaction term interacting percent urban population with a dummy variable that identifies countries with substantial urban slum populations and used it as a variable. The logic behind this interaction term is to capture the different association that urban development in developing regions with substantial slum populations might have with both life expectancy and carbon dioxide emissions.

The health effects of poverty and slum conditions are well researched [8, 9]. This research demonstrates that the prevalence of slums in developing nations is associated with higher infant and child mortality rates and lower life expectancy for adults, as well as a variety of other health problems [37, 38, 39, 40, 41]. By including this interaction effect, we are assessing whether the slope between urbanization and CIWB will be significantly different in developing countries with substantial slum populations than it is in countries that have urbanized without slums.

## Results

In model 1, we assess the relationship between urbanization (measured as the percentage of individuals living in urban areas) and CIWB, and control for theoretically relevant correlates of CIWB (total population, GDP per capita, percent of population between 15 and 64, and the percent of population over 65), finding that the percent of individuals living in urban areas has a positive and statistically significant relationship to nations’ CIWB through time. To build on model 1, in model 2 we assess whether the relationship between urbanization and CIWB is non-linear and create a quadratic term for percent urban populations, finding that it is not. In model 3, we create a dummy variable that uniquely identifies developing nations with slums populations and interact this variable with percent of individuals living in urban areas. The coefficient for this variable specifically displays the relationship of a proportional increase in the percentage of individuals living in urban areas within developing nations that have slums populations. Additionally, the coefficient for percent urban population in model 3 shows the association that developed countries that do not have substantial slum populations have on CIWB. The findings in model 3 indicate that for developing nations with slum populations the association between urbanization and CIWB is substantially lower than it is in all other nations included in our model.

**Fig 1:**
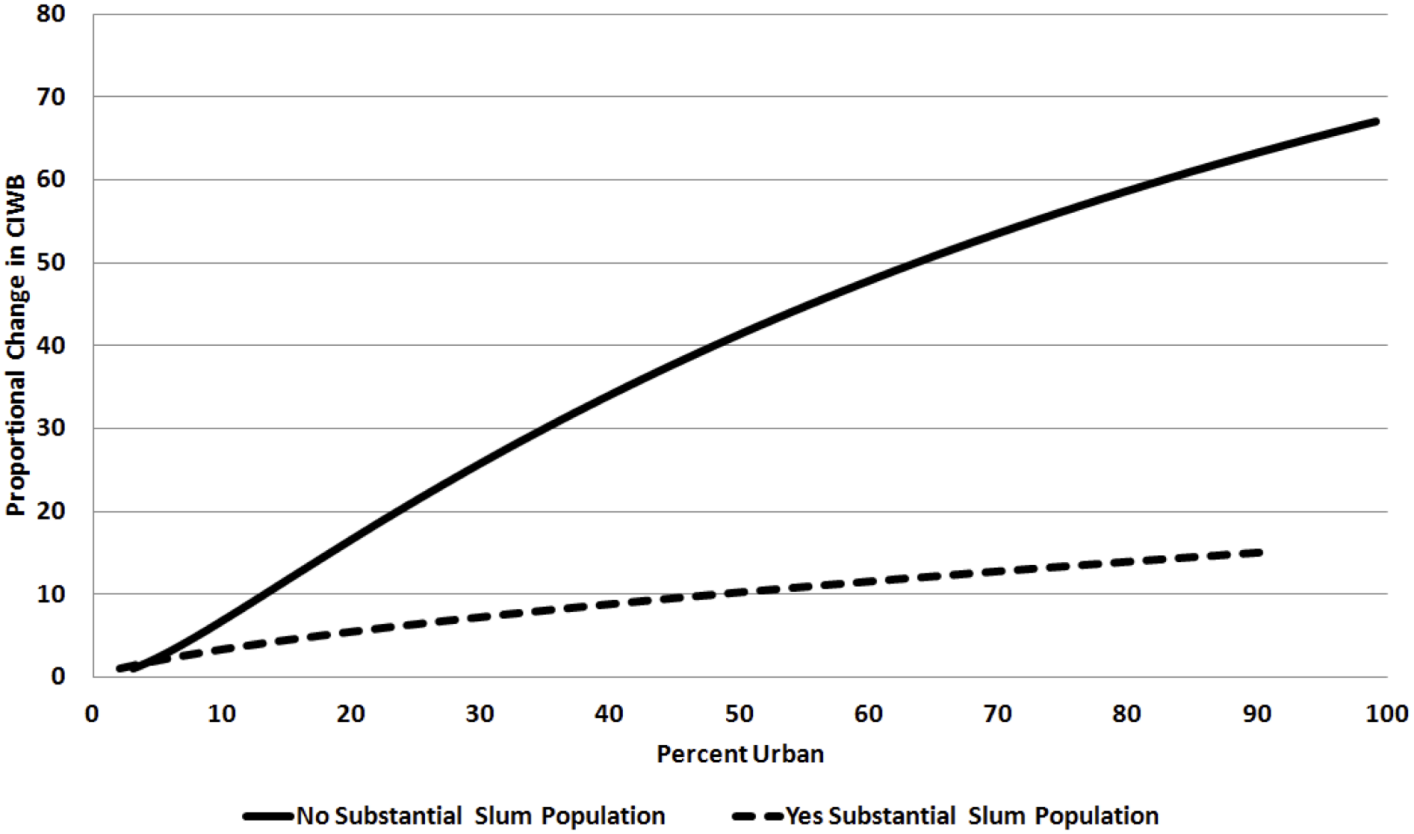
Dotted and solid lines represent, respectively, countries with and without substantial slum populations. To draw the graph in Fig. 1, we first used slope estimates from Model 4 to get predicted values of CIWB (within the range of observations) and then exponentiated these values. Second, we identified a baseline, which equals the unlogged predicted value of the CIWB at the minimum level of urbanization for countries with and without substantial spopulations, respectively, around 2% urban and around 3% urban. Third, we divided the unlogged predicted value of the CIWB for each (higher) level of urbanization by this baseline, yielding a ratio equal to the proportional change in CIWB as urbanization increases, compared to the baseline. For instance, with a substantial slum population, when the country is 50% urban, its predicted CIWB is roughly 10 times greater than the predicted CIWB at the minimum level of urbanization (again, around 2% urban). Without a substantial slum population, at 50% urban, the CIWB is approximately 40 times greater than the predicted CIWB at the minimum level of urbanization (around 3% urban). Compared to countries with substantial slum populations (dotted line), the proportional change in CIWB for countries without substantial slum populations (solid line) is much higher at lower levels of urbanization. While, at higher levels of urbanization, the rate of increase in CIWB for countries without substantial slum populations begins to slow down, there is no turning point at which the CIWB begins to decrease. Moreover, as we also see from the estimates in Model 4, having a substantial slum population actually moderates the association between urbanization and CIWB, although the relationship is more approximately linear.

In model 4, we find that the quadratic term for the variable representing the percent of individuals living in urban areas and its quadratic term are significant at a .05 test. Additionally, we find that the variable for interaction effect of percent of individuals living in urban populations and developing nations with slums populations are each significant at a .05 test. This suggests that (1) the relationship between percent urban population and CIWB is significantly different in developing countries that have slum populations than all other nations, and (2) that the relationship between percent urban population and CIWB in both developing nations that have slum populations and all other nations is non-linear. However, because the quadratic terms for both percent urban population and the interaction effect of developing nations with slum populations are significant, it is easiest to interpret their relationship to CIWB graphically. As can be seen in Fig 1, in nations with no slum populations, CIWB rises rapidly with urbanization, but begins to slow down at higher levels. Conversely, in nations with slum populations it appears that the relationship between CIWB and urbanization is more linear. When considering the breadth of literature that has contrasted urban growth in the developed and developing world, this finding is not at all that surprising, as it simply suggests that urban growth in developing countries with slums have a less intense association with CIWB, as well as a more linear trajectory. What is perhaps most interesting about these results is that growth in CIWB slightly attenuates as urban populations increase in all nations. Although, it should be stressed that this attenuation is very small through the range of values where this relationship was observed.

## Discussions

Here we find that the rate of increase in CIWB for countries without substantial slum populations begins to slow down at higher levels of urbanization and the association between urbanization and CIWB is much smaller in countries with substantial slum populations. One potential explanation for the findings presented here is that, in developing nations with substantial slum populations, urban growth has a smaller association to processes that produce CO_2_ emissions, such as energy consumption and the manufacturing of impervious surface area, than it does in nations with no slums. However, this also suggest that urban growth has a smaller positive association with life expectancy at birth in nations with substantial slum populations than in nations with no slums.

Previous research has also suggested that greater levels of urban density are associated with declines in counterfactual energy use [42], which one reviewer suggested is due to lower travel distances and less demand for heating because of the synergy effects of buildings. Though we are unable to incorporate such an insight into our modeling approach here, we note that another possible explanation of the reduced association of urban development on CIWB is that slums are often more densely populated, and have access to less safe and reliable infrastructure than other urban areas [43], which might lead to reduced resource use and emissions. Though examining the validity of these potential causes of reduced CIWB in developing nations with slum populations is beyond the scope of this study, we note that in either of these cases the reduced CIWB in nations with substantial slum populations is the result of unequal development patterns across nations [40]. This phenomenon presents a unique and difficult challenge for policy makers interested in global development and sustainability. Namely, such policy makers must concern themselves with finding means of promoting urban development plans and policies that drastically increase growth in life expectancy at birth relative to emissions in more developed countries, while also finding ways to encourage greater access to energy use and significant gains in life expectancy in those nations that do have substantial slum populations. Such actions, though difficult, would likely lead to a notable reduction in the gap between the relationship of urbanization and CIWB in nations with slum populations and those without substantial slum populations, and would represent an important step toward the achievement of global sustainable development.

Though it can be assumed that because we created an interaction term that specifically distinguishes between countries with slums and countries that are developed and/or have no slums that the differences observed in this analysis are a result of slums, we caution against such an interpretation. It would be inappropriate to argue that this is the only difference between urban development in countries that have slum populations and those that do not. One benefit to observing the association of rises in all urban populations, and not just substantial slum populations, however, is that it does allow our model to capture some of the other differences that exist between urban development across these groups.

We believe that the most notable contribution of this study is its ability to help further our understanding of the nuanced relationship between urban growth, well-being, and carbon dioxide emissions. Similar to previous studies in this area, we demonstrate the complexity of urban development by finding that there is not a linear relationship between CIWB and urban population growth in developing countries that have slum populations, and in countries that do not fit this specific criterion. The present study also shows the need for future research and policies aimed at reducing carbon emissions, while simultaneously increasing human well-being, to be mindful of the contrast between urbanization in developing nations that have slums and other countries. As mentioned above, in this study we are unable to directly examine the relationship between slum-patterned urban development and CIWB, or to compare this relationship directly with non-slum-patterned urban development’s relationship to CIWB, and we caution against such an interpretation. Here, our focus has been on the differences in the association between CIWB and urban development patterns in developing nations with substantial slum populations relative to nations without substantial slum populations as a whole, and not, per se, on the differences between slums and non-slum urban areas. However, we encourage future research to address the issue of slum development patterns and sustainability more directly. Finally, we note that, based on the observed trajectory of nations with and without slum populations, urbanization alone does not appear as though it is effective at reducing growth in CIWB. According to our findings, even as countries reach 100 percent urbanization the attenuation of CIWB is still fairly small (this is even more the case for nations with slum populations). If the current trajectory of urban growth continues, deepening our understanding how urbanization influences well-being and environmental sustainability will be increasingly crucial. The findings presented here offer a glimpse into how researchers can observe these relationships in a way that captures some of the differences in the forms of urbanization that can be seen across the globe.

## Methods

We constructed a Prais-Winsten regression model with panel-corrected standard errors (PCSE), allowing for disturbances that are heteroskedastic and contemporaneously correlated across panels [44]. We employ a Prais-Winsten regression model with panel corrected standard errors instead of using a lagged dependent variable, which is a common alternative used with cross sectional data, because we are conceptualizing urbanization as a proxy for a variety of other factors. To this end, we assess the association of urbanization in specific years because we are less concerned with the direct association of individuals moving into urban areas, which a lagged dependent variable may be better at capturing, and more interested in the broader implications of trends in urban development over time.

We use nations as our unit of analysis, and include year and country specific intercepts to control for potential heterogeneity that is temporally invariant within nations, and cross-sectionally invariant in time periods. Additionally, we correct for AR (1) disturbances within panels, treating the AR(1) process as common to all panels because there is no theoretical reason to assume the process is panel specific [5]. Our country level data were obtained from the World Bank’s Development Indicators [45]. The data are annual observations from 1960–2013. We included cross-sectional time series data for all nations where data was available. There are a number of missing values for specific countries in our dataset, with the minimum number of time periods observed in the dataset for any particular country being four.

To construct our dependent variable, we employ World Bank, World Development Indicators [6], data on anthropogenic carbon dioxide emissions per capita, and average life expectancy at time of birth within nations. All data used in this analysis can be found in its raw form in S1 File. We place anthropogenic carbon dioxide emissions per capita, which captures emissions from the burning of fossil fuels and the manufacture of cement measured in kilo tons, as our numerator in the CIWB ratio. Additionally, we use average life expectancy as our denominator. Doing so enables us to examine the change in a nation’s anthropogenically produced carbon dioxide emissions in relation to that nation’s change in average life expectancy.

As in previous research concerning well-being and environmental intensity [6, 12, 31, 32, 33, 46], we find that the coefficient of variation for carbon dioxide emissions per capita is much higher than the coefficient of variation for life expectancy. Specifically, the coefficient of variation for carbon dioxide emissions per capita is 2, while the coefficient of variation for life expectancy is 0.18. The result of the notable difference in these two coefficients of variation is that changes in the CIWB within any nation can potentially be the outcome of changes in the value of carbon dioxide emissions per capita. To prevent this, we follow previous research in adding a constant to the value of carbon dioxide emissions per capita in order to hold the coefficient of variation in the numerator and denominator equivalent to one another. The constant that allows for the two coefficients of variation to be made equal in our analysis is 0.04. Thus, our calculation for CIWB is as follows:

$$CIWB=[{(CO}_{2}PC+0.04)/LE]*100$$

We include GDP per capita, measured in constant 2005 US dollars, as an independent variable in order to account for the level of economic development within nations. Previous research has found economic development to be a critical factor in country-level CIWB outcomes [16, 20]. Specifically, economic development increases both life expectancy at birth and emissions. Additionally, we include the percent of population between 15 and 64, and the percent of population over 65 as control variables. Each of these variables uniquely relate to CIWB. For example, previous work has found that they each have a significant impact on carbon emissions and energy use, because the age structure of a population predicts the productive capacity of a nation. Furthermore, the portion of individuals between the ages of 15 and 64 relative to the portion of individuals over the age of 65 are strong indicators of the overall life expectancy of in nations. Finally, we control for the association between total population and CIWB, which, although is not a common variable used in analyses of CIWB, is found to significantly influence CIWB. Controlling for total population allows our model to account for the relationship between the relative size of a population and CIWB. Note, because population is not commonly controlled for as an independent variable in CIWB analyses, we produced additional models that excluded population as an independent variable to assess the robustness of our model. In these models, we found that not controlling for total population had little effect on the coefficients for our other independent variables (no changes in P-values and no changes in the direction of coefficients), but did slightly increase our R-squared.

Following York and Colleagues [8], we log all variables in the model in order to reflect the elastic relationship between anthropogenic drivers and the ratio between carbon dioxide emissions and well-being. The result of this is that findings represent the proportional change in CIWB for every one-percent change in a given predictor variable.

The form of the Prais-Winsten regression model with panel corrected standard errors including all independent variables used in Table 1 can be expressed as follows:

$$\begin{array}{l} \begin{array}{l} \text{ln}\left(ciwb_{it}\right)=\\ \beta_{0}+\beta_{1}\text{ln}\left(percent\;urban_{it}\right)+\\ \beta_{2}\text{ln}\left(dummy\;for\;nations\;with\;slums_{it}\right)\left(percent\;urban_{it}\right)+\beta_{3}\text{ln}\left(population_{it}\right)+\\ \beta_{4}\text{ln}\left(GDP\;per\;capita_{it}\right)+\beta_{5}ln\left(percent\;population\;{15-65}_{it}\right)+\\ \beta_{6}\text{ln}\left(percent\;population\;over\;65_{it}\right)+\beta_{7}\left(year\;1960_{it}\right)\;\ldots +\beta_{9}\left(year\;2013_{t}\right)+u_{i}+e_{it} \end{array} \end{array}$$

Where “CIWB_*it*_”, our outcome of interest, represents the carbon intensity of well-being for nation *i* in year *t*; “Percent Urban_*it*_” indicates the percent of the population the is urbanized in country *i* during year *t*; "Dummy Variable for Nation with Slums_*it*_” indicates whether nation *i* has slum patterned urbanization in year *t*; "Population_*it*_" is a control for total population size of nation *i* in time *t*; “GDP per capita_*it*_" represents nation *i’s* GDP per capita in year *t*, “Percent Population 15-65_*it*_” indicates the percent of the productive population during time *t* in country *i*; “Percent Population Over 65_*it*_” represents the percent of the population that is typically unproductive in country *t* during year *t*; “year_*t*_” is a control for period specific effects; u_*i*_ is a control for nation specific, non-contemporaneous, effects; and e_*it*_ is the residual term for nation *i* in period *t*.

**Table 1:** Prais-Winsten regression models with panel-corrected standard errors of influences on carbon intensity of well-being.

	Model 1 Coef. (SE)	Model 2 Coef. (SE)	Model 3 Coef. (SE)	Model 4 Coef. (SE)
**Percent urban population**	.823*** (.081)	.940*** (.242)	1.071*** (.116)	2.251*** (.407)
**Quadratic term for percent urban population**		**.-021** **(.045)**		-.182** (.028)
**Interaction term for percent urban population and slum control**			-.334** (.117)	-1.427** (.464)
**Interaction term for quadratic of percent urban population and slum control**				.162* (.073)
**Population**	1.369*** (.076)	1.376*** (.079)	1.417*** (.082)	1.396*** (.084)
**GDP per capita**	.686*** (.042)	.689*** (.041)***	.673** (.041)	.675*** (.041)
**Percent of population age 15–65**	1.104*** (.686)	1.127*** (.2250)	1.013*** (.240)	1.021** (.247)
**Percent of population age 65 and over**	.285*** (.082)	.289*** (.085)	.294*** (.085)	.295*** (.085)
**R-squared**	**.9681**	**.9682**	**.9682**	**.9683**
**N (Nations/total data points)**	**182/6871**	**182/6871**	**182/6871**	**182/6871**

*** p < .001

** p < .01

* p < .05

Note: Including country specific and time specific intercepts in panel corrected standard errors models inflates R-squared estimates because these variables account for any unobserved year to year changes and unobserved changes across countries, which is most of the variation in our model. As a result, including additional variables only slightly changes R-squared estimates because most of the variation is accounted for in the country and year specific variables.

Note: Analyses are across nations 1960–2013. All variables are in natural logarithmic form. All models include year and country intercepts (not shown).

## Appendix 1

### Nations with slums

Angola, Argentina, Bangladesh, Belize, Benin, Bolivia, Brazil, Burkina Faso, Burundi, Cambodia, Cameroon, Central African Republic, Chad, Chile, China, Colombia, Comoros, Democratic Republic of The Congo, Republic of The Congo, Costa Rica, Cote d’Ivoire, Dominican Republic, Ecuador, Egypt, El Salvador, Equatorial Guinea, Ethiopia, Gabon, Ghana, Grenada, Guatemala, Guinea, Guinea-Bissau, Guyana, Haiti, Honduras, India, Indonesia, Iraq, Jordan, Kenya, Lao, Lebanon, Lesotho, Madagascar, Malawi, Mali, Mexico, Mongolia, Morocco, Mozambique, Namibia, Nepal, Nicaragua, Niger, Nigeria, Pakistan, Panama, Paraguay, Peru, Philippines, Rwanda, Saudi Arabia, Senegal, Sierra Leone, South Africa, Sudan, Suriname, Tanzania, Thailand, Togo, Trinidad and Tobago, Turkey, Uganda, Vietnam, Zambia, Zimbabwe.

### Nations without slums

Afghanistan, Albania, Algeria, Antigua and Barbuda, Armenia, Aruba, Australia, Austria, Azerbaijan, The Bahamas, Bahrain, Barbados, Belarus, Belgium, Bhutan, Bosnia and Herzegovina, Botswana, Brunei Darussalam, Bulgaria, Cabo Verde, Canada, Croatia, Cuba, Cyprus, Czech Republic, Denmark, Djibouti, Eritrea, Estonia, Fiji, Finland, France, Gambia, Georgia, Germany, Greece, Hong Kong, Hungary, Iceland, Iran, Ireland, Israel, Italy, Japan, Kazakhstan, Kiribati, Republic of Korea, Kuwait, Kyrgyz Republic, Latvia, Liberia, Libya, Lithuania, Luxembourg, Macao, Macedonia, Malaysia, Malta, Mauritania, Mauritius, Micronesia, Moldova, Montenegro, Netherlands, New Zealand, Norway, Oman, Papua New Guinea, Poland, Portugal, Qatar, Romania, Russian Federation, Samoa, Sao Tome and Principe, Serbia, Seychelles, Singapore, Slovak Republic, Slovenia, Solomon Islands, Spain, Sri Lanka, St. Lucia, St Vincent and The Grenadines, Swaziland, Sweden, Switzerland, Syria, Tajikistan, Timor-Leste, Tonga, Tunisia, Turkmenistan, Ukraine, United Arab Emirates, United Kingdom, United States, Uruguay, Uzbekistan, Vanuatu, Venezuela, West Bank and Gaza, Yemen.

## Supporting information

S1 File Raw data. This file contains all data used to perform the analyses presented by the authors above.
(CSV)
